# Nodal Error behind Discrepancies between Coupled Cluster and Diffusion Monte Carlo

**DOI:** 10.1021/acs.jctc.6c00882

**Published:** 2026-07-20

**Authors:** S. Lambie, P. López Ríos, D. Kats, A. Alavi

**Affiliations:** † 28326 Max Planck Institute for Solid State Research, Heisenbergstraße 1, Stuttgart 70569, Germany; ‡ Yusuf Hamied Department of Chemistry, University of Cambridge, Lensfield Road, Cambridge CB2 1EW, United Kingdom

## Abstract

The small magnitude and long-range character of noncovalent interactions pose a significant challenge for computational quantum chemical and electronic structure methods alike. State-of-the-art coupled cluster (CC) theory and benchmark-grade diffusion Monte Carlo (DMC) are ideally positioned to tackle these problems, but concerning differences between both methods have been reported in numerous studies of the interaction energy of noncovalently bound dimers. Given that the basic theoretical frameworks underpinning both methods are exact in principle, the error must arise from one or several of the approximations required to make the calculations computationally tractable. Here, we carry out a rigorous and systematic examination of the effect of each of these approximations using the acetic acid dimer and water–peptide systems as convenient testing grounds. Thanks to the use of stringently optimized backflow wave functions, we are able to find that the significant discrepancies are dominated by the fixed-node error incurred by the Slater-Jastrow DMC result, while errors in the CC calculations do not significantly alter the result. This finding, likely applicable to other hydrogen-bonded systems, helps establish that CC should be regarded as the benchmark for these systems and can potentially guide the search for pragmatic solutions to the fixed-node problem in the future.

## Graphical abstract

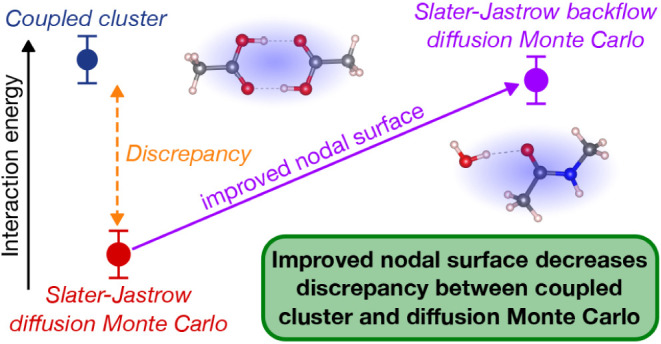

## 1 Introduction

The quality and relevance of a computational model lie in its ability to reproduce and predict the experimentally observed phenomena. However, obtaining reliable experimental data that can be directly compared to computational results is a complicated and fraught endeavor, especially for noncovalent interactions. In an experiment, the effects of temperature and solvent are embedded in any measurement and must be disentangled from the measurement itself; a nontrivial process that introduces errors that are difficult to quantify. By contrast, theoretical calculations establish the interaction energy between two rigid molecules at 0 K in the gas phase, with the comparison to experiment being further exacerbated by the typically small magnitude of these interactions. High-quality quantum chemical calculations approximating the exact full configuration interaction (FCI) result in the complete basis set (CBS) limit, providing crucial benchmark results to which the quality of other theoretical models can be established.

In quantum chemistry, coupled cluster (CC) theory^1,2^ has long provided a systematically improvable, hierarchical approach for moving toward the exact solution of the Schrödinger equation for molecules of moderate sizes as more excitations are included in the cluster operator. In turn, the diffusion quantum Monte Carlo (DMC) method has emerged among electronic structure methods as one of the most accurate approaches to solving the Schrödinger equation for systems with several hundred electrons. As computational resources have become more powerful over the past few decades, CC has become applicable to larger system sizes, while DMC has seen improvements in the quality with which moderately sized systems can be described. Comparisons of the two methods are, therefore, of great interest,^3–6^ and several recent studies in the literature have focused on disagreements found between CC and DMC in weakly interacting systems, for which DMC is expected to be particularly well-suited.^7–9^

Both CC theory and the DMC method are, in principle, capable of exactly solving the Schrödinger equation,^10^ but in practice, both require the use of a variety of approximations. In the case of CC theory, the cluster operator is typically truncated at the singles, doubles, and perturbative triples (CCSD­(T)) level,^11^ which provides a very good balance of accuracy and computational cost compared to CCSDT­(Q) and CCSDTQ^12^ for a majority of chemical properties.^13,14^ However, the use of finite basis sets and the truncation of the cluster operator introduce errors into the calculation that grow as system size increases. The DMC method requires controlled approximations, such as the use of finite time steps and walker populations, and uncontrolled approximations, such as the use of pseudopotentials and the localization approximation they require. However, the bulk of the error incurred by DMC is ultimately from the fixed-node approximation, whereby the DMC energy depends on the quality of the nodes of a trial wave function that guides the stochastic sampling process.

The finding of significant discrepancies between the two methods for noncovalent interaction energies^7,8^ stimulated a flurry of additional studies investigating the possible sources of disagreement.^15–21^ The issue was initially thought to be restricted to large, dispersion-bound systems, but substantial deviations were also found for small H-bound systems.^9^ To date, the origin of the discrepancy between the two state-of-the-art methods has not been definitively identified. Here, we focus on the two case-study examples of the 16-atom acetic acid (AcOH) dimer and 15-atom water–peptide system, corresponding to systems 20 and 4 of the hydrogen-bonded portion of the S66 set, respectively;^22^ see insets in Figure 1. For the AcOH dimer, a discrepancy of about 0.8 kcal mol^–1^ has been reported between the DMC value of −20.17(7) kcal mol^–1^ and the canonical CC value of −19.39(2) kcal mol^–1^,^9^ while for the water–peptide system, the difference between the DMC result of −8.58(7) kcal mol^–1^ and the CC result of −8.20(2) kcal mol^–1^ is about 0.4 kcal mol^–1^.^9^ The AcOH dimer and water–peptide systems thus provide a wonderful opportunity for probing sources of error between the two methods, thanks to the large energy discrepancies and small system sizes.

**Figure 1:**
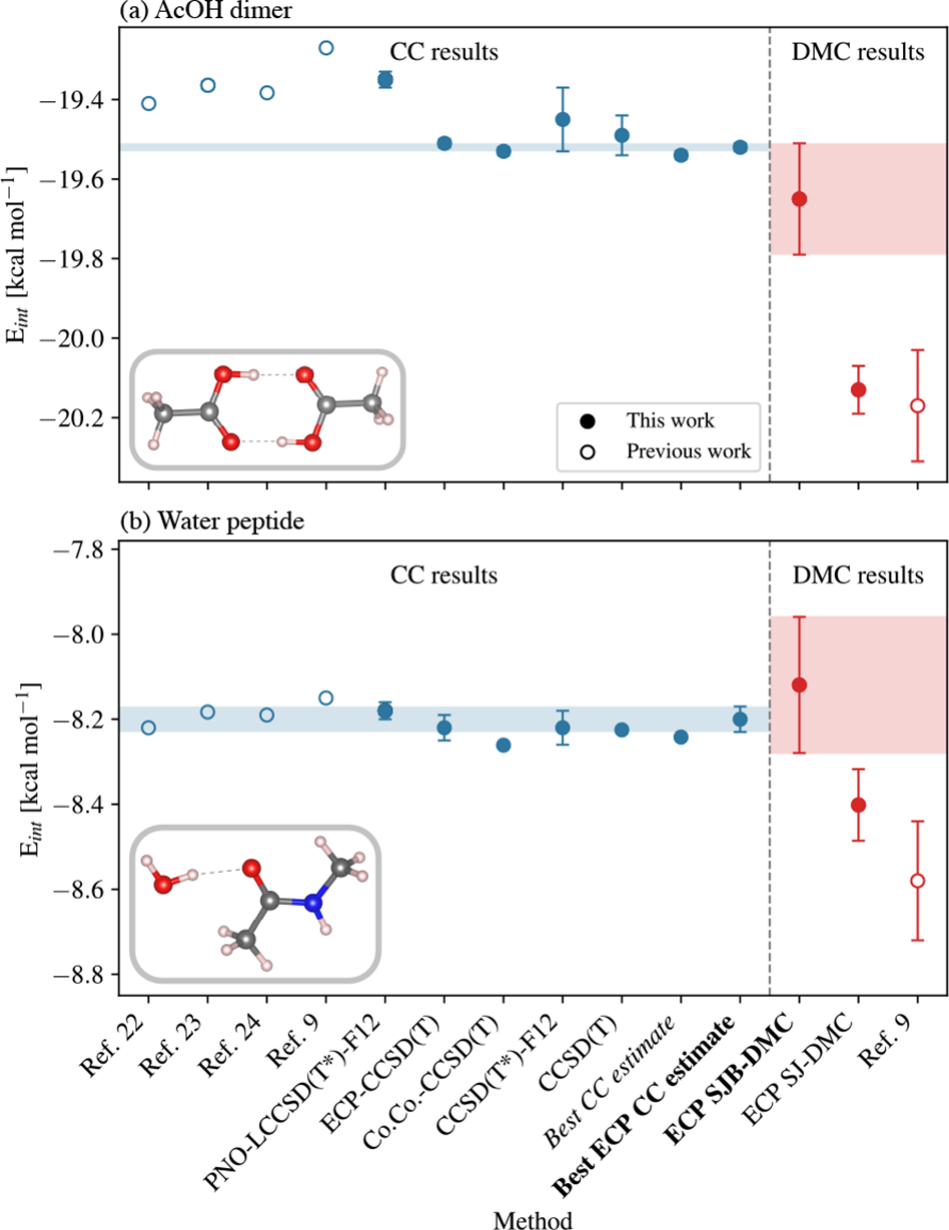
Interaction energy of (a) the AcOH dimer and (b) the water–peptide dimer obtained using CC theory (blue) and DMC (red). The interaction energies that allow for the most direct comparison between each methodology (bold), with a “two-sigma” confidence interval, are shown as a shaded area. For the CC calculations, 0.5CP-corrected values are reported, with nonstatistical error bars extending from the CP-uncorrected to the CP-corrected result; note that reference CC values are plotted as single points. The error bars on the DMC values are purely statistical in nature, representing 95% (two-sigma) confidence intervals, and include optimization uncertainty where pertinent; see the text. The insets show the structures considered, with C atoms in gray, O atoms in red, H atoms in pink, and N atoms in blue.

## 2 Coupled Cluster Results

In CC theory, “gold standard” results are obtained by truncating the CC operator at the CCSD­(T) level, which provides an excellent balance of accuracy and computational cost due to a systematic cancellation of errors.^13,14,26^ However, a beyond-“gold” standard is required for benchmarking quantum chemical methods, taking into consideration all possible sources of error in CCSD­(T). We have studied the convergence of CC results with respect to the basis set (Section 2.1), electronic excitations (Section 2.2), local implementation (Section 2.3), and CC operator (Section 2.4).

### 2.1 Approaching the Complete Basis Set Limit

All basis sets are finite and, as a result, incomplete, meaning that both basis set superposition error (BSSE)^27^ and basis set incompleteness error (BSIE)^28^ require careful management. The counterpoise (CP) correction is the most common approach for dealing with BSSE,^29^ which allows the CBS limit to be approached more smoothly and quickly than with uncorrected (raw) values.^30,31^ Raw and CP-corrected results typically provide a lower and upper bound, respectively, for the CBS value, within a specific CC operator truncation,^31^ and for this reason, we report our CC results as the arithmetic mean of the raw and CP-corrected values, plus/minus half of the full CP correction, so that the “two-sigma” interval, which corresponds to the 95% confidence interval for statistical standard errors, covers the full CP range. This simplifies comparison with DMC, where error bars occur naturally, but bear in mind that our CC “error bars” are not statistical in nature. In the CBS limit, both the BSIE and BSSE vanish, along with the need for CP corrections.

Correlation energy is slowly convergent with basis-set size,^32–36^ and basis sets of at least augmented triple-ζ (aVTZ) size are essential for CC results to be considered reliable for noncovalent interactions.^37^ For this reason, the largest computationally affordable basis sets are used in conjunction with techniques to approximate the CBS limit, such as focal-point (FP) approaches,^31,38^ CBS extrapolations,^30,34^ and explicitly correlated F12 methods.^39^

FP approaches^31,38^ exploit the fact that the total energy of a post-Hartree–Fock method can be decomposed into the Hartree–Fock (HF) component and various correlation energy contributions calculated using basis sets of different sizes, taking advantage of the fact that the energy offset between methods is fairly consistent. The FP approach can then be coupled further with a CBS extrapolation^30,34^ so that the correlation energy component of the calculation can be obtained in smaller basis sets and extrapolated to the CBS limit. Explicitly correlated methods significantly improve basis set convergence by including geminal functions that rely on the interelectronic distance into the wave function ansatz.^39,40^ The (T) treatment does not contain any F12 terms,^41^ and therefore, the (T) energy must be scaled (see Section 5), resulting in the CCSD­(T*)-F12 method. Performance of CCSD­(T*)-F12 with aVTZ and aVQZ basis sets is known to be comparable to CBS-extrapolated canonical CCSD­(T)/aV­{Q,5}­Z results for noncovalent interactions.^42^ Here, the CCSD­(T*)-F12 results agree with the CCSD­(T) results (Table 1), to which it can be most directly compared, determining that both the explicitly correlated method and CBS-extrapolated results converge toward the CBS limit for the interaction energy of the AcOH dimer and water–peptide system at the CCSD­(T) level of theory. For the AcOH dimer, we additionally test the truncated heavy-atom-only augmented^43^ and density-fitted^44,45^ basis sets with minimal changes to the results; see Section S1 of the Supporting Information.

**Table 1:** Interaction Energy of the AcOH Dimer from CC Theory in kcal mol^–1^, Expressed as the 0.5CP-Corrected Value Plus/Minus Half of the Total CP Correction Except Where Otherwise Stated^a^

This work			
		*E*_int_ [kcal mol^–1^]	
In text-referral	Treatment	(AcOH)_2_	Water–peptide
CCSD(T)	DF-HF/aV5Z + CCSD(T)/aV{T,Q}Z	–19.49(5)	–8.225(5)
CCSD(T*)-F12	DF-HF-F12/aV5Z + CCSD(T*)-F12/aVQZ	–19.45(8)	–8.22(4)
Co.Co.-CCSD(T)	DF-HF/aCV5Z + CCSD(T)/aCV{T,Q}Z	–19.53(1)	–8.261(3)
ECP-CCSD(T)	DF-HF/aV5Z-eCEPP + CCSD(T)/aV{T,Q}Z-eCEPP	–19.51(1)	–8.22(3)
PNO-LCCSD(T*)-F12	DF-HF-CABS/aV5Z + PNO-LCCSD(T*)-F12/aV5Z	–19.35(2)	–8.18(2)
*Best CC estimate*	HF/aCV5Z + CCSD(T)/aCV{T,Q}Z + ΔSVD-DC-CCSDT+/aVTZ	*–19.54(1)*	*–8.242(3)*
**Best ECP CC estimate**	DF-HF/aV5Z-eCEPP + CCSD(T)/aV{T,Q}Z-eCEPP+ΔSVD-DC-CCSDT+/aVTZ	**–19.52(1)**	**–8.20(3)**

Reference values			
		*E*_int_ [kcal mol^–1^]	
In text-referral	Treatment	(AcOH)_2_	Water–peptide
ref 23	HF/aV{T,Q}Z + MP2/aV{T,Q}Z + ΔCCSD(T)/haV{D,T}Z (CP)	–19.41	–8.22
ref 24	HF-CABS/V5Z-F12 + MP2-F12/aV{T,Q}Z-F12 + ΔCCSD(F12*)/VTZ-F12 + Δ(T)/haV{D,T}Z	–19.364(5)	–8.18(1)
ref 25	HF-CABS/aVQZ-F12+ MP2/aV{T,Q}Z-F12 + ΔCCSD(*)/haV{D,T}Z+Δ(T)/haV{T,Q}Z (mixed)	–19.38	–8.19
ref 9	ref 24 + CCSD(cT)-fit (mixed)	–19.27	–8.15

a A bold typeface is used for our best CC theory AcOH dimer interaction energy with ECPs.

In general, we find that our CC results for the AcOH dimer and the water–peptide system are in good agreement with the literature (Table 1).^23–25^ However, the results of the present study can be expected to be more accurate than previously reported values, thanks in part to our use of larger basis sets. Additionally, the previously reported values use the FP approach extensively—that is, they make two key assumptions that could introduce an undefined error into the results. First, all reference values assume that the difference in energy between lower-order methods, such as MP2 and CCSD­(T), is constant and unchanging as the size of the basis set is increased. Second, they assume that these energetic differences are converged in small basis sets, such as the heavy-atom augmented double-triple-ζ extrapolation ΔCCSD­(T) energetic correction, used in ref 23. While the FP approach is generally applicable, and introduces small errors, these errors are not necessarily systematic^31^ and are relevant on the scale of interest for the noncovalent interactions reported in the present study. As such, it is imperative to control all sources of error in the coupled cluster calculations, and by calculating the correlation energy in large basis sets with minimal usage of the FP approach, we control these additional sources of error in the present calculations. It should also be noted that some of the previous studies referenced here reported individual interaction energies containing a specific amount of CP correction, and are therefore comparable with the outer limits of our “two-sigma” confidence intervals.

### 2.2 Core Electron Treatments

The frozen-core approximation is routinely used in CC calculations, but its effect on the results is rarely considered. In the present study, calculations invoke the frozen-core approximation unless otherwise stated. Alternative treatments of the core region exist, in particular, the explicit inclusion of core correlation in the calculation (Co.-Co.-CCSD­(T)) through the use of correlation-consistent core–valence basis sets, aCVX Z, X = T, Q, where the C indicates that the basis set is suitable for correlating core electrons,^46^ or the core can be treated using effective core potentials (ECP-CCSD­(T)), as commonly used in DMC calculations; specifically, we use energy-consistent correlated electron pseudopotentials (eCEPPs).^47^

We have performed calculations of the AcOH dimer and water–peptide system with all three treatments of the core, and we find that a very small stabilization, on the order of 0.04 kcal mol^–1^ arises as a result of including core correlation in the calculation. Therefore, we conclude that core treatment is unlikely to be responsible for the significant discrepancy between the CC and DMC results.

### 2.3 Local Coupled Cluster

Local methods^48–50^ are designed to reach larger system sizes with high accuracy and are often used to obtain CCSD­(T)/CBS reference values for noncovalent interaction energies of large molecules.^7,8,21,51,52^ Here, we focus on the most accurate of the local methods, PNO-LCCSD­(T*)-F12,^50^ where the combination of local and scaled F12 methods is highly effective at reducing the basis-set and domain errors.^50,53–55^ However, both basis-set and local errors increase as the system size grows,^50,56^ and therefore, the fidelity of local methods to the canonical result is expected to deteriorate with an increasing system size.^50,52^ While the reduced scaling offered by local methods is not required to simulate the systems considered in this study due to their small size, we test this method for its performance compared to the canonical result to better understand possible sources of error.

The PNO-LCCSD (T*)-F12 calculations are a small underestimation of the CCSD­(T*)-F12 results (Table 1), as expected by the method developers.^50,57^ While the low basis set error of local methods^58–61^ would appear to indicate a low error on local CC values, we advise that confidence intervals be estimated conservatively for local calculations, extending beyond the basis set error, until the extrapolation of local results to the canonical limit is well established.^62^

### 2.4 Beyond Gold Standard Coupled Cluster

For molecular dimers, it has been found that CCSD­(T) regularly outperforms CCSDT^13,14,20,63,64^ and that CCSDT­(Q) and CCSDTQ typically produce results that are converged with respect to the cluster operator for noncovalent interactions.^13^ However, the perturbative treatment of excitations in CC theory causes diverging results in the metallic limit.^17,19,65^ Since the discrepancy between DMC and CC results was first reported for increasingly large dispersion-bound C-based molecules^7–9^ with diminishing HOMO–LUMO gaps,^66–68^ the lack of convergence with respect to the truncation of the CC operator must be considered as a possible source of error.

Even for the small systems considered in this study, the *N* ^9^ scaling of CCSDT­(Q) means that canonical calculations with large basis sets are unattainable. Therefore, post-CCSD­(T) corrections can be obtained by approximating the CC operator through the use of CCSD­(cT)^19^ or rank-reduced distinguishable cluster (SVD-DC-CCSDT+)^69–72^ methods, or by using very small, truncated basis sets to carry out canonical CCSDT­(Q) calculations.^64^

Each of these approaches has its own limitations. CCSD­(cT)^19^ approximates CCSDT, which is known to be somewhat inaccurate for noncovalent interactions.^13,14,20,63,64^ SVD-DC-CCSDT+^69^ enables larger basis sets to be used but is a new method that has currently only been benchmarked against the A24 data set.^73,74^ Additional benchmarking of the SVD-DC-CCSDT+ method for the AcOH dimer and water–peptide system is shown in the Supporting Information. Canonical CCSDT­(Q) results^64^ require the use of small basis sets, implying that these corrections cannot be expected to be converged with respect to basis set size.^75^ Nevertheless, these three approaches result in small post-CCSD­(T) corrections of, for the AcOH dimer, 0.12 kcal mol^–1^, −0.01 kcal mol^–1^ and 0.055 kcal mol^–1^, and for the water–peptide system of 0.05 kcal mol^–1^, 0.019 kcal mol^–1^ and 0.012 kcal mol^–1^, respectively, for fitted CCSD­(cT),^9^ SVD-DC-CCSDT+/aVTZ, and CCSDT­(Q)/VDZ­(d,s).^64^ Interestingly, all of the results considering higher-order excitations in the cluster operator than CCSD­(T) have either negligible effects or reduce the magnitude of the CC result, thereby increasing the discrepancy between the DMC and CC results. We, therefore, conclude that the discrepancy with DMC is highly unlikely to be sourced from truncation of the CC operator.

### 2.5 Other Considerations and the Best Coupled Cluster Estimate

Our best CC estimates for the interaction energy of the AcOH dimer and water–peptide system using CC-based methodologies are −19.54(1) kcal mol^–1^ and −8.242(3) kcal mol^–1^, respectively. These calculations improve upon previously reported values by using larger basis sets for the correlation energy component of the calculations, considering excitations from the deep core, and an approximate post-CCSD­(T) correction. Other possible sources of error in our best estimate could arise from scalar relativistic effects and diagonal Born–Oppenheimer corrections, however, these are known to be negligibly small for light atoms,^76^ such as C, N, O, and H, found in (AcOH)_2_ and the water–peptide system, and are also absent from the DMC calculations.

Since none of the approximations employed in CC theory are able to explain the discrepancy with the DMC results, we turn to examining possible sources of error arising from the approximations involved in the DMC calculations. To directly compare CC and DMC, we take the ECP-CCSD­(T) result and add a post-CCSD­(T) correction to obtain our “best ECP CC estimate.”

## 3 Diffusion Quantum Monte Carlo Results

### 3.1 Slater–Jastrow Results

We have run DMC calculations of the AcOH, (AcOH)_2_, water, peptide, and water–peptide systems using the Slater–Jastrow (SJ) trial wave function and ECPs to represent ionic cores, namely, eCEPPs,^47^ with which we recover ECP SJ-DMC results within uncertainty of those reported in ref 9; see Table 2 and Figure 1.

**Table 2:** DMC Interaction Energy of the AcOH Dimer and the Water–Peptide System, in kcal mol^–1^ ^a^

This work		
	*E*_int_ [kcal mol^–1^]	
Method	(AcOH)_2_	Water–peptide
AE SJ-DMC	–20.29(16)	-
ECP SJ-DMC	–20.13(6)	–8.40(8)
**ECP SJB-DMC**	**–19.65(14)**	**–8.12(16)**

Reference values		
	*E*_int_ [kcal mol^–1^]	
Method	(AcOH)_2_	Water–peptide
ECP SJ-DMC^9^	–20.17(14)	–8.58(14)

a Uncertainties shown represent 95% (2-Sigma) confidence intervals. A bold typeface is used for our best DMC interaction energy.

There are various potential sources of error in the DMC results. DMC calculations are performed at finite time steps τ, and the resulting energies must be extrapolated to zero time step, as we report in Section S2 the Supporting Information. Population control bias is typically negligible and can be removed by extrapolation along with time-step bias,^77^ which we have done. We have verified that the error incurred by the use of ECPs is negligible on the scale of interest by computing the all-electron (AE) SJ-DMC interaction energy of the AcOH dimer, reported in Table 2, which agrees within uncertainty with its ECP counterpart. We have tested using distinct Jastrow parameters for symmetry-inequivalent atoms,^78^ but we find that this does not change the interaction energy estimate for (AcOH)_2_. The source of the remaining error in DMC is the mismatch between the nodes of the trial wave function and those of the exact wave function, which is referred to as the fixed-node error. Note that BSIE can be regarded as part of the fixed-node error in DMC; our use of the relatively large aVTZ basis set should provide a good starting point for this source of fixed-node error.^16,79^

### 3.2 Beyond Slater–Jastrow Nodes Backflow Results

The use of multideterminant expansions is a popular approach for obtaining beyond-SJ nodes to study electronic excitations of small molecules,^80–90^ but it suffers from significant size-consistency issues that preclude the calculation of accurate interaction energies, especially for the system sizes of interest here, so we have instead chosen to apply backflow^91–95^ to Ψ_S_. In the Slater-Jastrow-backflow (SJB) wave function, the arguments of the single-particle orbitals are replaced with quasiparticle coordinates that depend on the positions of all other electrons, smoothly altering the shape of the SJ nodal surface; see Section S2 of the Supporting Information for further details. SJB-DMC is often capable of removing about half of the fixed-node error in the SJ-DMC energy.^77,81,82^ The use of backflow incurs a significant increase in the computational cost of DMC calculations, so it is typically not used for large systems, but the expense is manageable for the systems studied here when ECPs are used. The topical, very flexible neural-network wave functions^96,97^ incorporate Jastrow-, backflow-, orbital-, and multideterminant-like degrees of freedom, but we have opted against using these because they incur potentially greater costs, require specialized workflows and hardware, and preclude straightforward comparisons with SJ-DMC.

Variational quantum Monte Carlo (VMC) based optimization of Jastrow factors is usually performed without considering the presence of statistical uncertainties in the resulting parameters arising from the stochastic nature of the optimization process. This is because the Jastrow factor parameters in the SJ wave function do not affect the value of (all-electron) SJ-DMC energies at τ = 0, but there are scenarios where wave function parameters significantly affect expectation values,^98–100^ as is the case of the DMC energy in the presence of backflow. Ignoring optimization uncertainty can thus result in the perception that SJB-DMC energies behave erratically and are inconsistent across systems.

The optimization uncertainty on the SJB-DMC energy σ_opt_ depends on the number of real-space configurations *n*_opt_ used in VMC-based correlated-sampling optimization via $\sigma_{\mathrm{opt}}^{2}=\alpha /n_{\mathrm{opt}}$ , where α is a system-dependent unknown, at sufficiently large *n*_opt_; see Section S2 of the Supporting Information. For a given *n*_opt_, the optimization uncertainty on the SJB-DMC energy can be evaluated by running multiple independent random instances of optimization at that sample size, each followed by a SJB-DMC run. It is therefore possible to perform this procedure at a trial value of *n*_opt_ to obtain σ_opt_ in order to determine α, and thus be able to identify the value of *n*_opt_ necessary to obtain any given target accuracy

To verify that the relationship between σ_opt_ and *n*_opt_ is applicable in practice, we have computed the optimization uncertainty on the SJB-DMC energy of the AcOH monomer, $\sigma_{\mathrm{opt}}^{\mathrm{AcOH}}$ , for several values of *n*_opt_. In Figure 2a the energies from individual instances of optimization at each sample size are shown, and Figure 2b is a log–log plot of $\sigma_{\mathrm{opt}}^{\mathrm{AcOH}}$ as a function *n*_opt_, which confirms that the optimization uncertainty remains proportional to $n_{\mathrm{opt}}^{-1/2}$ in the entire range of *n*_opt_ tested.

**Figure 2:**
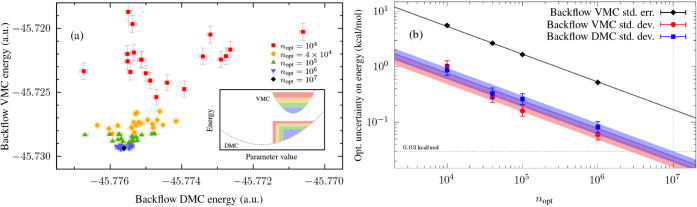
(a) Scatter plot of the SJB-VMC energy as a function of SJB-DMC energy of the ECP AcOH monomer for 20 independent random instances of wave function optimization at each of four optimization sample sizes, *n*_opt_, and for the final optimization performed at *n*_opt_ = 10^7^; note the lack of visible correlation between VMC and DMC energies and the evident mismatch of the location of the VMC and DMC energy minima, schematically represented in the inset. (b) Resulting uncertainty (1-sigma confidence interval) on the SJB-VMC and SJB-DMC energy arising from the stochastic nature of optimization as a function of *n*_opt_, computed as the standard deviation of the corresponding energies in Figure 2a, in log–log scale. The target optimization uncertainty and chosen sample size are shown as dotted lines, and solid lines are linear fits in log–log scale to the data points using a fixed slope of −1/2. Shaded areas and error bars represent 95% (two-sigma) confidence intervals. Also shown is the (average) standard error on the mean value of the *n*_opt_ local energies used in optimization, which is an order of magnitude greater than the optimization uncertainty on the backflow VMC energy: correlated-sampling optimizers can determine the location of the energy minimum to much better accuracy than the statistical resolution of the local energy sample mean would suggest.

Using the average value of α obtained from these data, we pick a final optimization sample size of $n_{\mathrm{opt}}^{\mathrm{AcOH}}=n_{\mathrm{opt}}^{{\left(\mathrm{AcOH}\right)}_{2}}=10^{7}$ to achieve a target optimization uncertainty on the interaction energy of σ_opt_ = 0.071 kcal mol^–1^, set so as to obtain a total uncertainty on the interaction energy below 0.10 kcal mol^–1^ (see Figure 2b); note that $\sigma_{\mathrm{opt}}^{\mathrm{AcOH}}=\sigma_{\mathrm{opt}}/\sqrt{6}\approx 0.029\;\mathrm{kcal}\;{\mathrm{mol}}^{-1}$ , as discussed in Section S2 of the Supporting Information.

For the heterogeneous water–peptide system, we choose to split the target optimization uncertainty of σ_opt_ = 0.071 kcal mol^–1^ in proportion to the square root of the number of electrons in each of the systems, to keep it in line with the stochastic uncertainty, and thus arrive at $\sigma_{\mathrm{opt}}^{\mathrm{water}}=0.023\;\mathrm{kcal}\;{\mathrm{mol}}^{-1}$ , $\sigma_{\mathrm{opt}}^{\mathrm{peptide}}=0.045\;\mathrm{kcal}\;{\mathrm{mol}}^{-1}$ , and $\sigma_{\mathrm{opt}}^{\mathrm{w.-p.}}=0.050\;\mathrm{kcal}\;{\mathrm{mol}}^{-1}$ . In this case, we evaluate the optimization uncertainty at a single value of *n*_opt_ = 3 × 10^4^, and we thus determine target optimization sample sizes of $n_{\mathrm{opt}}^{\mathrm{water}}=1.1\times 10^{7}$ , $n_{\mathrm{opt}}^{\mathrm{peptide}}=10^{6}$ and $n_{\mathrm{opt}}^{\mathrm{w.-p.}}=7.5\times 10^{6}$ .

Our final SJB-DMC interaction energies of −19.65(14) kcal mol^–1^ for (AcOH)_2_ and −8.12(16) kcal mol^–1^ for water–peptide, given in Table 2 and plotted in Figure 1, are in excellent agreement with our best ECP CC energy estimates of −19.52(1) kcal mol^–1^ and −8.20(3) kcal mol^–1^, respectively, strongly supporting the hypothesis that the main cause of the disagreement between CC and SJ-DMC results in H-bonded systems is the fixed-node error.

While the backflow correction to the interaction energies is of the order of tenths of a kcal mol^−1^, the backflow corrections to the total energies of the (AcOH)_2_ and water−peptide systems are −20.08(6) and −14.22(5) kcal mol^−1^, respectively, which are substantial corrections relative to other nodal improvement methods (see, e.g., ref 101), as discussed in the Supporting Information. However, the backflow correction to the total DMC energy is still only a fraction of fixed-node error present at the SJ-DMC level, implying that the fixed-node error is orders of magnitude larger than the scale of interest, and hence obtaining subchemical accuracy requires an exquisite level of cancellation of errors which SJ-DMC does not guarantee. In DMC workflows involving optimization of the nodes, the “variance matching” technique has been shown to successfully promote error cancellation,^89,90,102,103^ and in this vein we have computed the VMC variance of the local energy associated with our backflow wavefunctions, with well-defined confidence intervals obtained with the tail-regression estimator^104^ as provided by the TREAT utility.^105^ We find that the VMC variance of the local energy of each dimer differs marginally from the sum of those of the corresponding monomers, in such a way that an exactly variance-matched description would, if anything, shift the SJB-DMC interaction energy slightly closer to the CC result, leaving our conclusions unchanged.

## 4 Discussion and Conclusions

We have considered various potential sources of error in CC and DMC calculations of the AcOH dimer and water–peptide system and find that the lifting of various approximations does not significantly alter the CCSD­(T) result.

In both of the case-study examples considered in this work, the use of backflow, which reduces the fixed-node error, brings the magnitude of the DMC interaction energy toward the CC result, in such a way that the two methods are now effectively in concordance with one another. From this, we come to the conclusion that the fixed-node error in the SJ-DMC calculations is responsible for the vast majority of the disagreement between the methods reported in the literature for this class of systems. This implies that the inconsistencies in the nodes of the HF wave function (which the SJ wave function inherits) between the monomer and dimer, while arguably small given the weakness of the interaction, suffice to put DMC at a disadvantage with respect to CC. The application of backflow will undoubtedly be crucial in resolving the discrepancies between CC and DMC in dispersion-bound systems, too.

In order to obtain meaningful backflow results, we have given explicit consideration to the uncertainty introduced by the stochastic nature of the optimization process, which accounts for the largest share of the uncertainty on our final result. Establishing the magnitude of this optimization uncertainty is crucial in ensuring that DMC energies obtained using trial wave functions with stochastically optimized nodes are well-defined quantities not affected by uncontrolled noise.

Using backflow in the way we have in our present work is not necessarily a practical or affordable solution to the fixed-node error problem of the SJ-DMC. Our SJB-DMC calculations have used 2.9 million core-hours on an AMD EPYC cluster to obtain the interaction energy of (AcOH)_2_ and 1.5 million for the water–peptide system, while their SJ-DMC counterparts cost 0.42 and 0.27 million core-hours, respectively. On top of this, we have used an unrefined approach with conservative choices of run parameters to establish the optimal value of *n*_opt_, adding 1.2 and 3.4 million core-hours to the cost of the (AcOH)_2_ and water–peptide calculations, respectively. Despite its costliness, the backflow ansatz is trivially size-consistent, unlike multideterminant expansions, and the optimization protocol proposed in the present work prevents this property from being adversely affected by the stochastic optimization process. This approach allows backflow to be deployed in cases where beyond-SJ nodes are important, prompting further work to make the use and optimization of backflow more affordable in practice.

## 5 Computational Details

In keeping with previous works, we utilize the S66 geometries^22^ for the AcOH dimer (system 20) and the water–peptide system (system 4) throughout the study, as available from the Benchmark Energy and Geometry Database;^101^ see Section S4 of the Supporting Information.

When calculating the interaction energy, no relaxation effect of the monomers is taken into account. Intermolecular energies are calculated with the supermolecular approach; for the AcOH dimer as

1 $$E_{\mathrm{int}}=E_{\mathrm{dimer}}-2E_{\mathrm{monomer}}$$

and for the water–peptide system as

2 $$E_{\mathrm{int}}=E_{\mathrm{dimer}}-E_{\mathrm{monomer 1}}-E_{\mathrm{monomer 2}}$$

### 5.1 Canonical CC Calculations

CCSD­(T) calculations were carried out with MOLPRO 2022.3.^102,103^

CBS extrapolations are calculated using the form of Helgaker^34^ and Halkier,^30^ as follows

3 $$E\left(XY\right)=\frac{\left(X^{3}E_{X}-Y^{3}E_{Y}\right)}{X^{3}-Y^{3}}$$

where *X* and *Y* describe the cardinal number of the basis set, and *E*_*X*_ and *E*_*Y*_ are the total correlation energies in each basis set, respectively. Basis sets employed are the augmented correlation-consistent Dunning^104^ aug-cc-pV*X*Z basis sets with X = T, Q, or 5, abbreviated to aV*X*Z. The aVTZ and aVQZ extrapolation is, for example, then denoted as aV­{T, Q}­Z. Basis set extrapolations are only carried out on the correlation energy, which is more slowly convergent with BS size, while the HF energy is not extrapolated due to its faster convergence with BS size.^30,34^ Where density fitting is employed, JKFIT^44^ and MPFIT^45^ of the same cardinal number as the atomic orbital basis set are used.

For the explicitly correlated calculations, the F12b explicit correlation method^39,40^ is used, as it systematically converges to the CBS limit.^39^ The scaling of the (T) energy is carried out as

4 $$E_{\left(T^{*}\right)}=E_{\left(T\right)}\frac{E_{\mathrm{MP2‐F12}}}{E_{\mathrm{MP2}}}$$

where all energies are correlation energies, of (T), MP2, and MP2-F12, to ultimately, give a (T*) correlation energy. The scaling factor of the dimer is used for the monomer scaling in both the raw and CP-corrected calculations to ensure consistency between calculations and in keeping with previous recommendations.^50^ It is unclear how these methods should be extrapolated to the CBS limit, and therefore, no basis set extrapolations are carried out for either the canonical or local explicitly correlated methods.

For the Co.Co.-CCSD­(T) calculations, the core region was set to zero, and the core-correlated correlation-consistent basis sets, aug-cc-pCV*X*Z,^46^ were used. For the ECP-CCSD­(T) calculations, the energy-consistent correlated electron pseudopotentials (eCEPPs) of Trail and Needs were employed.^47^

### 5.2 PNO-LCCSD­(T*)-F12b

Local CC calculations were carried out using (T*)-scaled, explicitly correlated pair natural orbital local CCSD­(T) (PNO-LCCSD­(T*)-F12b).^57,102,103^ Tight domain and pair thresholds^50,105^ were used, and complete auxiliary basis set (CABS) corrections^39,40^ of the HF energy were utilized. The energy threshold for the local CCSD pair natural orbital domains was set to 0.997, as recommended for the intermolecular interactions.

### 5.3 SVD-DC-CCSDT+

The SVD-DC-CCSDT+ ^69–72,106,107^ method is an approximation to CC theory that uses the distinguishable cluster approximation to remove selected exchange terms from the CC amplitude equations, combined with an SVD treatment of the cluster amplitudes.^108–110^ SVD-DC-CCSDT+ calculations are carried out in ElemCo.jl.^111^

An SVD amplitude threshold of 10^–6^ was used in conjunction with the SVD-(T) correction scheme, as described in ref 74 to obtain the SVD-DC-CCSDT+ energies. Importantly, the SVD-DC-CCSDT+ method is used to obtain a post-CCSD­(T) correction to the energy in the aVTZ basis set, calculated within the frozen core approximation as

$$\Delta \mathrm{SVD‐DC‐CCSDT+/aVTZ}=E_{\mathrm{corr.}}\left(\mathrm{SVD‐DC‐CCSDT+/aVTZ}\right)-E_{\mathrm{corr.}}\left(\mathrm{CCSD}\left(T\right)/\mathrm{aVTZ}\right)$$ 5

### 5.4 DMC Calculations

Wave function optimization and DMC runs have been performed using the casino code.^77^ Within DMC we handle the eCEPPs^47^ using the T-move scheme^112,113^ as DMC localization approximation, and we use the size-consistent local-energy limiting Green’s function modifications of Zen et al.^114^ to enable accurate, efficient extrapolations to zero time step.^115,116^ Note that, by contrast with the calculations reported in ref 9, the single-particle orbitals in our SJ wave function are Gaussian expansions of HF orbitals using the aug-cc-pVTZ basis set^104^ instead of B-spline^117^ re-expansions of local-density-approximation or Perdew–Burke–Ernzerhof plane-wave orbitals, which we cusp-correct in AE calculations,^118^ and we optimize our Jastrow^119,120^ and backflow^95^ parameters using linear least-squares energy minimization^121,122^ instead of unreweighted variance minimization.^123^ Further details are reported in Section S2 of the Supporting Information.

## Supplementary Material







## Data Availability

The authors declare that all data supporting the findings of this study are included in the paper and are available within the paper and its Supporting Information files. Additional data are available upon reasonable request from the authors.

## Notes

Additional computational details, benchmarking, and verification of the methods employed in this work (PDF)
Raw energy data and processing scripts used for the deffusion Monte Carlo calculations (ZIP)
Benchmark interaction energy calculations, F12 scaling factors, and post-CCSD­(T) corrections for the acetic acid dimer and water–peptide systems (XLSX)

## References

[1] Cížek J. (1966). On the correlation
problem in atomic and molecular systems. Calculation of wavefunction
components in Ursell-type expansion using quantum-field theory methods. J. Chem. Phys..

[2] Paldus J., Čížek J., Shavitt I. (1972). Correlation problems
in atomic and molecular systems. IV. Extended coupled-pair many-electron
theory and its application to the BH_3_ molecule. Phys. Rev. A.

[3] Maten S., Lüchow A. (2001). On the accuracy of the fixed-node diffusion quantum
Monte Carlo method. J. Chem. Phys..

[4] Mella M., Anderson J. B. (2003). A brute force test
of accuracies for He_2_ and He-LiH. J. Chem. Phys..

[5] Dubecký M. (2014). Quantum Monte Carlo for noncovalent interactions An efficient protocol
attaining benchmark accuracy. Phys. Chem. Chem.
Phys..

[6] Dubecký M. (2013). Quantum
Monte Carlo describe noncovalent interactions with subchemical
accuracy. J. Chem. Theory Comput..

[7] Al-Hamdani Y. S., Nagy P. R., Zen A., Barton D., Kállay M., Brandenburg J. G., Tkatchenko A. (2021). Interactions between large molecules
pose a puzzle for reference quantum mechanical methods. Nat. Commun..

[8] Villot C., Ballesteros F., Wang D., Lao K. U. (2022). Coupled cluster
benchmarking of large noncovalent complexes in L7 and S12L as well
as the C_60_ dimer, DNA-ellipticine and HIV-indinavir. J. Phys. Chem. A.

[9] Shi B. X., Della Pia F., Al-Hamdani Y. S., Michaelides A., Alfè D., Zen A. (2025). Systematic
discrepancies
between reference methods for non-covalent interactions within the
S66 dataset. J. Chem. Phys..

[10] Witte J., Goldey M., Neaton J. B., Head-Gordon M. (2015). Beyond energetics
Geometries of nonbonded molecular complexes as metrics for assessing
electronic structure approaches. J. Chem. Theory
Comput..

[11] Raghavachari K., Trucks G. W., Pople J. A., Head-Gordon M. (1989). A fifth-order
perturbation comparison of electron correlation theories. Chem. Phys. Lett..

[12] Kállay M., Gauss J. (2005). Approximate treatment of higher excitations
in coupled-cluster theory. J. Chem. Phys..

[13] Rezáč J., Šimová L., Hobza P. (2013). CCSD­[T] describes noncovalent interactions
better than the CCSD­(T), CCSD­(TQ) and CCSDT methods. J. Chem. Theory Comput..

[14] Rezáč J., Hobza P. (2013). Describing noncovalent interactions beyond the common approximations
How accurate is the “gold standard” CCSD­(T) at the complete
basis set limit?. J. Chem. Theory Comput..

[15] Nakano K., Sorella S., Alfé D., Zen A. (2024). Beyond single-reference
fixed-node approximation in ab initio diffusion Monte Carlo using
antisymmetrised geminal power applied to systems with hundreds of
electrons. J. Chem. Theory Comput..

[16] Nakano K., Shi B. X., Alfé D., Zen A. (2025). Basis set incompleteness
errors in fixed-node diffusion Monte Carlo calculations on non-covalent
interactions. J. Chem. Theory Comput..

[17] Benali A., Shin H., Heinonen O. (2020). Quantum Monte
Carlo benchmarking
of large noncovalent complexes in the L7 benchmark set. J. Chem. Phys..

[18] Gray M., Herbert J. M. (2024). Assessing the domain-based
local pair natural orbital
(DLPNO) approximation for non-covalent interactions in sizable supramolecular
complexes. J. Chem. Phys..

[19] Schäfer T., Irmler A., Gallo A., Grüneis A. (2024). Understanding
discrepancies of wavefunction theories for large molecules. Nat. Commun..

[20] Lambie S., Kats D., Usyvat D., Alavi A. (2024). On the applicability
of CCSD­(T) for dispersion interactions in large conjugated systems. J. Chem. Phys..

[21] Lao K. U. (2024). Canonical
coupled cluster binding benchmark for nanoscale noncovalent complexes
at the hundred-atom scale. J. Chem. Phys..

[22] Rezáč J., Riley K. E., Hobza P. (2011). S66 A well-balanced database of benchmark
interaction energies relevant to biomolecular structures. J. Chem. Theory Comput..

[23] Řezáč J., Riley K. E., Hobza P. (2011). Extensions of the S66 data set More
accurate interaction energies and angular displaced nonequilibrium
geometries. J. Chem. Theory Comput..

[24] Kesharwani M. K., Karton A., Sylvetsky N., Martin J. M. L. (2018). The S66 non-covalent
interactions benchmark reconsidered using explicitly correlated methods
near the basis set limit. Aust. J. Chem..

[25] Nagy P. R., Gyevi-Nagy L., Lörincz B. D., Kállay M. (2022). Pursuing the
basis set limit of CCSD­(T) non-covalent interaction energies for medium-sized
complexes Case study on the S66 compilation. Mol. Phys..

[26] Helgaker T., Ruden T. A., Jørgensen P., Olsen J., Klopper W. (2004). A priori calculation
of molecular properties to chemical accuracy. J. Phys. Org. Chem..

[27] Liu B., McLean A. D. (1973). Accurate calculation of the attractive interaction
of two ground state helium atoms. J. Chem. Phys..

[28] Davidson E. R., Feller D. (1986). Basis set selection
for molecular calculations. Chem. Rev..

[29] Boys S. F., Bernardi F. (1970). The calculation of
small molecular interactions by
the differences of separate total energies. Some procedures with reduced
errors. Mol. Phys..

[30] Halkier A., Klopper W., Helgaker T., Jørgensen P., Taylor P. R. (1999). Basis set convergence of the interaction
energy of
hydrogen-bonded complexes. J. Chem. Phys..

[31] Burns L. A., Marshall M. S., Sherrill C. D. (2014). Comparing
counterpoise-corrected,
uncorrected, and averaged binding energies for benchmarking noncovalent
interactions. J. Chem. Theory Comput..

[32] Hobza P., Selzle H. L., Schlag E. W. (1996). Potential
energy surface for the
benzene dimer. Results of ab initio CCSD­(T) calculations show two
nearly isoenergetic structures T-shaped and parallel displaced. J. Phys. Chem..

[33] Shaw R. A., Hill J. G. (2018). Midbond basis functions for weakly bound complexes. Mol. Phys..

[34] Helgaker T., Klopper W., Koch H., Noga J. (1997). Basis-set
convergence
of correlated calculations on water. J. Chem.
Phys..

[35] Steele R.
P., DiStasio R. A., Head-Gordon M. (2009). Non-covalent interactions with dual-basis
methods Pairings for augmented basis sets. J.
Chem. Theory Comput..

[36] Kodrycka M., Patkowski K. (2019). Platinum, gold and silver standards of intermolecular
energy calculations. J. Chem. Phys..

[37] Řezáč J., Hobza P. (2016). Benchmark calculations of interaction energies in noncovalent complexes
and their applications. Chem. Rev..

[38] Marshall M. S., Burns L. A., Sherrill C. D. (2011). Basis set
convergence of the coupled-cluster
correction Best practices for benchmarking non-covalent interactions
and attendant revision of the S22, NBC10, HBC6 and HSG databases. J. Chem. Phys..

[39] Knizia G., Adler T. B., Werner H.-J. (2009). Simplified
CCSD­(T)-F12 methods Theory
and benchmarks. J. Chem. Phys..

[40] Adler T. B., Knizia G., Werner H.-J. (2007). A simple and efficient CCSD­(T)-F12
approximation. J. Chem. Phys..

[41] Patkowski K. (2013). Basis set
converged weak interactions from conventional and explicitly correlated
coupled-cluster approach. J. Chem. Phys..

[42] Sirianni D. A., Burns L. A., Sherrill C. D. (2017). Comparison of explicitly correlated
methods for computing high-accuracy benchmark energies for noncovalent
interactions. J. Chem. Theory Comput..

[43] Marshall M. S., Sears J. S., Burns L. A., Breédas J.-L., Sherrill C. D. (2010). An error and efficiency analysis
of approximations
to Møller-Plesset perturbation theory. J. Chem. Theory Comput..

[44] Weigend F. (2002). A fully direct
RI-HF algorithm Implementation, optimized auxiliary basis sets, demonstration
of accuracy and efficiency. Phys. Chem. Chem.
Phys..

[45] Weigend F., Köhn A., Hättig C. (2002). Efficient use of the correlation
consistent basis sets in resolution of the identity MP2 calculations. J. Chem. Phys..

[46] Peterson K. A., Dunning T. H. (2002). Accurate correlation consistent basis sets for molecular
core-valence correlation effects The second row atoms Al-Ar and the
first row atoms B-Ne revisited. J. Chem. Phys..

[47] Trail J. R., Needs R. J. (2017). Shape and energy
consistent pseudopotentials for correlated
electron systems. J. Chem. Phys..

[48] Riplinger C., Sanhoefer B., Hansen A., Neese F. (2013). Natural triple excitations
in local coupled cluster calculations with pair natural orbitals. J. Chem. Phys..

[49] Nagy P. R., Samu G., Kállay M. (2018). Optimization
of the linear-scaling
local natural orbital CCSD­(T) method Improved algorithm and benchmark
applications. J. Chem. Theory Comput..

[50] Ma Q., Werner H.-J. (2018). Explicitly correlated
local coupled-cluster methods
using pair natural orbitals. WIREs Comput. Mol.
Sci..

[51] Nagy P. R. (2024). State-of-the-art
local correlation methods enable affordable gold standard quantum
chemistry for up to hundreds of atoms. Chem.
Sci..

[52] Hansen A., Knowles P. J., Werner H.-J. (2025). Accurate
calculation of noncovalent
interactions using PNO-LCCSD­(T)-F12 in Molpro. J. Phys. Chem. A.

[53] Jakubikova E., Rappé A. K., Bernstein E. R. (2006). Exploration
of basis set issues for
calculation of intermolecular interactions. J. Phys. Chem. A.

[54] Hill J. G., Platts J. A., Werner H.-J. (2006). Calculation of intermolecular interactions
in the benzene dimer using coupled- cluster and local electron correlation
methods. Phys. Chem. Chem. Phys..

[55] Krause C., Werner H.-J. (2012). Comparison of explicitly
correlated local coupled-cluster
methods with various choices of virtual orbitals. Phys. Chem. Chem. Phys..

[56] Altun A., Ghosh S., Riplinger C., Neese F., Bistoni G. (2021). Addressing
the system-size dependence of the local approximation error in coupled-cluster
calculations. J. Phys. Chem. A.

[57] Ma Q., Werner H.-J. (2018). Scalable electron
correlation methods. 5. Parallel
perturbative triples correction for explicitly correlated local coupled
cluster with pair natural orbitals. J. Chem.
Theory Comput..

[58] Hampel C., Werner H.-J. (1996). Local treatment of electron correlation
in coupled
cluster theory. J. Chem. Phys..

[59] Saebø S., Tong W., Pulay P. (1993). Efficient
elimination of basis set
superposition errors by the local correlation method Accurate *ab initio* studies of the water dimer. J. Chem. Phys..

[60] Runeberg N., Schütz M., Werner H.-J. (1999). The aurophilic attraction as interpreted
by local correlation methods. J. Chem. Phys..

[61] Schütz M., Rauhut G., Werner H.-J. (1998). Local treatment
of electron correlation
in molecular clusters Structures and stabilities of (H_2_O)*
_n_, n* = 2–4. J. Phys. Chem. A.

[62] Sorathia K., Tew D. P. (2020). Basis set extrapolation in pair natural orbital theories. J. Chem. Phys..

[63] Demovičová L., Hobza P., Rezáč J. (2014). Evaluation of composite schemes for
CCSDT­(Q) calculations of interaction energies of noncovalent complexes. Phys. Chem. Chem. Phys..

[64] Semidalas E., Boese A. D., Martin J. M. L. (2025). Post-CCSD­(T) corrections in the S66
noncovalent interactions benchmark. Chem. Phys.
Lett..

[65] Shepherd J. J., Grüneis A. (2013). Many-body
quantum chemistry for the electron gas Convergent
perturbative theories. Phys. Rev. Lett..

[66] Shen B., Tatchen J., Sanchez-Garcia E., Bettinger H. F. (2018). Evolution
of the optical gap in the acene series Undecacene. Angew. Chem., Int. Ed..

[67] Tönshoff C., Bettinger H. F. (2020). Pushing the limits of acene chemistry The recent surge
of large acenes. Chem.–Eur. J..

[68] Novoselov K. S. (2004). Electronic field effect in atomically thin
carbon films. Science.

[69] Rickert C., Usyvat D., Kats D. (2025). Tensor decomposed
distinguishable
cluster.I. Triples Decomposition. J. Chem. Phys..

[70] Kats D. (2014). The distinguishable
cluster approximation. II. The role of orbital relaxation. J. Chem. Phys..

[71] Kats D., Manby F. R. (2013). Communication The
distinguishable cluster approximation. J. Chem.
Phys..

[72] Kats D., Köhn A. (2019). On the distinguishable
cluster approximation for triple
excitations. J. Chem. Phys..

[73] Řezáč J., Dubecký M., Jurečka P., Hobza P. (2015). Extensions and applications
of the A24 dataset of accurate interaction energies. Phys. Chem. Chem. Phys..

[74] Lambie S., Rickert C., Kats D., Usyvat D., Alavi A. (2025). Benchmarking
distinguishable cluster methods to platinum standard CCSDT­(Q) non
covalent interaction energies in the A24 dataset. J. Chem. Phys..

[75] Smith D. G. A., Slawik P. J. M., Witek H., Patkowski K., Patkowski K. (2014). Basis set convergence of the post-CCSD­(T)
contribution
to noncovalent interaction energies. J. Chem.
Theory Comput..

[76] Karton A., Martin J. M. L. (2021). Prototypical *π-π* dimers
re-examined by means of high-level CCSDT­(Q) composite *ab initio* methods. J. Chem. Phys..

[77] Needs R. J., Towler M. D., Drummond N. D., López Ríos P., Trail J. R. (2020). Variational and diffusion quantum Monte Carlo calculations
with the CASINO code. J. Chem. Phys..

[78] Dubecký M., Jurečka P., Mitas L., Ditte M., Fanta R. (2019). Toward accurate
hydrogen bonds by scalable quantum monte carlo. J. Chem. Theory Comput..

[79] Dubecký M. (2017). Noncovalent
interactions by fixed-node diffusion Monte Carlo convergence of nodes
and energy differences vs gaussian basis-set size. J. Chem. Theory Comput..

[80] Filippi C., Umrigar C. J. (1996). Multiconfiguration wave functions
for quantum Monte
Carlo calculations of first row diatomic molecules. J. Chem. Phys..

[81] Brown M. D., Trail J. R., López Ríos P., Needs R. J. (2007). Energies
of the first row atoms from quantum Monte Carlo. arxiv.

[82] Seth P., López Ríos P., Needs R. J. (2011). Quantum Monte Carlo
study of the first-row atoms and ions. J. Chem.
Phys..

[83] Petruzielo F. R., Toulouse J., Umrigar C. J. (2012). Approaching
chemical accuracy with
quantum Monte Carlo. J. Chem. Phys..

[84] Morales M. A., McMinis J., Clark B. K., Kim J., Scuseria G. E. (2012). Multideterminant
wave functions in quantum Monte Carlo. J. Chem.
Theory Comput..

[85] Giner E., Assaraf R., Toulouse J. (2016). Quantum Monte
Carlo with reoptimised
perturbatively selected configuration-interaction wave functions. Mol. Phys..

[86] Scemama A., Benali A., Jacquemin D., Caffarel M., Loos P.-F. (2018). Excitation
energies from diffusion Monte Carlo using selected configuration interaction
nodes. J. Chem. Phys..

[87] Scemama A., Caffarel M., Benali A., Jacquemin D., Loos P.-F. (2019). Influence of pseudopotentials on excitation energies
from selected configuration interaction and diffusion Monte Carlo. Results Chem..

[88] Dash M., Moroni S., Scemama A., Filippi C. (2018). Perturbatively selected
configuration-interaction wave functions for efficient geometry optimization
in quantum Monte Carlo. J. Chem. Theory Comput..

[89] Dash M., Feldt J., Moroni S., Scemama A., Filippi C. (2019). Excited states
with selected configuration interaction-quantum Monte Carlo Chemically
accurate excitation energies and geometries. J. Chem. Theory Comput..

[90] Dash M., Moroni S., Filippi C., Scemama A. (2021). Tailoring
CIPSI expansions
for QMC calculations of electronic excitations The case study of thiophene. J. Chem. Theory Comput..

[91] Feynman R. P., Cohen M. (1956). Energy spectrum of the excitations
in liquid helium. Phys. Rev.

[92] Lee M. A., Schmidt K. E., Kalos M. H., Chester G. V. (1981). Green’s function
Monte Carlo method for liquid ^3^He. Phys. Rev. Lett..

[93] Kwon Y., Ceperley D. M., Martin R. M. (1998). Effects of backflow
correlation in
the three-dimensional electron gas Quantum Monte Carlo study. Phys. Rev. B.

[94] Holzmann M., Ceperley D. M., Pierleoni C., Esler K. (2003). Backflow correlations
for the electron gas and metallic hydrogen. Phys. Rev. E.

[95] López
Ríos P., Ma A., Drummond N. D., Towler M. D., Needs R. J. (2006). Inhomogeneous backflow transformations in quantum Monte
Carlo calculations. Phys. Rev. E.

[96] Pfau D., Spencer J. S., Matthews A. G. D. G., Foulkes W. M. C. (2020). Ab initio solution
of the many-electron Schrödinger equation with deep neural
networks. Phys. Rev. Res..

[97] Hermann J., Schätzle Z., Noé F. (2020). Deep-neural-network solution of the
electronic Schrödinger equation. Nat.
Chem..

[98] Haupt J. P., Hosseini S. M., López
Ríos P., Dobrautz W., Cohen A., Alavi A. (2023). Optimizing Jastrow factors
for the transcorrelated method. arxiv.

[99] Filip M.-A., Christlmaier E. M. C., Haupt J. P., Kats D., López
Ríos P., Alavi A. (2025). Deterministic optimization
of Jastrow factors. arxiv.

[100] Spink G. G., López Ríos P., Drummond N. D., Needs R. J. (2016). Trion formation in a two-dimensional
hole-doped electron
gas. Phys. Rev. B.

[101] Řezáč J. (2008). Quantum chemical benchmark
energy and geometry database for molecular clusters and complex molecular
systems (begdb.org) A users manual and examples. Collect. Czech. Chem. Commun..

[102] Werner H.-J. (2012). Molpro A general-purpose quantum chemistry
program package. WIREs Comput. Mol. Sci..

[103] Werner H.-J., Knowles P. J., Manby F. R., Black J. A., Doll K., Heßelmann A., Kats D., Köhn A., Korona T., Kreplin D. A. (2020). The Molpro quantum chemistry
package. J. Chem. Phys..

[104] Dunning
Jr T. H. (1989). Gaussian basis sets for use in correlated molecular
calculations. I. The atoms boron through neon and hydrogen. J. Chem. Phys..

[105] Werner H.-J., Hansen A. (2023). Accurate calculation of isomerization
and conformational energies of larger molecules using explicitly correlated
local coupled cluster methods in Molpro and ORCA. J. Chem. Theory Comput..

[106] Rishi V., Valeev E. F. (2019). Can the distinguishable cluster approximation
be improved systematically by including connected triples?. J. Chem. Phys..

[107] Schraivogel T., Kats D. (2021). Accuracy of
the distinguishable cluster
approximation for triple excitations for open-shall molecules and
excited states. J. Chem. Phys..

[108] Hino O., Kinoshita T., Bartlett R. J. (2004). Singular value decomposition
applied to the compression of *t*
_3_ amplitude
for the coupled cluster method. J. Chem. Phys..

[109] Kinoshita T., Hino O., Bartlett R. J. (2003). Singular
value decomposition
approach for the approximate coupled-cluster method. J. Chem. Phys..

[110] Lesiuk M. (2020). Implementation
of the coupled-cluster method with single,
double and triple excitations using tensor decomposition. J. Chem. Theory Comput..

[111] Kats, D. ; Schraivogel, T. ; Hauskrecht, J. ; Rickert, C. ; Wu, F. ElemCo.jl Julia program package for electron correlation methods; elem.co.il, 2024.

[112] Casula M. (2006). Beyond the
locality approximation in the standard diffusion
Monte Carlo method. Phys. Rev. B.

[113] Casula M., Moroni S., Sorella S., Filippi C. (2010). Size-consistent
variational approaches to nonlocal pseudopotentials Standard and lattice
regularized diffusion Monte Carlo methods revisited. J. Chem. Phys..

[114] Zen A., Sorella S., Gillan M. J., Michaelides A., Alfè D. (2016). Boosting the accuracy and speed of
quantum Monte Carlo
Size consistency and time step.,Phys. Rev. B.

[115] Lee R. M., Conduit G. J., Nemec N., López
Ríos P., Drummond N. D. (2011). Strategies for improving the efficiency
of quantum Monte Carlo calculations. Phys. Rev.
E.

[116] Vrbik J., Rothstein S. M. (1986). Optimal
spacing and weights in diffusion
Monte Carlo. Int. J. Quantum Chem..

[117] Alfè D., Gillan M. J. (2004). Efficient localized
basis set for
quantum Monte Carlo calculations on condensed matter Phys. Rev. B.

[118] Ma A., Towler M. D., Drummond N. D., Needs R. J. (2005). Scheme for adding
electron–nucleus cusps to Gaussian orbitals. J. Chem. Phys..

[119] López Ríos P., Seth P., Drummond N. D., Needs R. J. (2012). Framework for constructing
generic Jastrow correlation
factors. Phys. Rev. E.

[120] Drummond N. D., Towler M. D., Needs R. J. (2004). Jastrow correlation
factor for atoms, molecules, and solids. Phys.
Rev. B.

[121] Toulouse J., Umrigar C. J. (2007). Optimization of
quantum Monte Carlo
wave functions by energy minimization. arxiv.

[122] Umrigar C. J., Toulouse J., Filippi C., Sorella S., Hennig R. G. (2007). Alleviation of the fermion-sign problem
by optimization
of many-body wave functions. Phys. Rev. Lett..

[123] Schmidt K. E., Moskowitz J. W. (1990). Correlated
Monte Carlo wave functions
for the atoms He through Ne. J. Chem. Phys..

