# Supplementary material for: Nodal Error behind Discrepancies between Coupled Cluster and Diffusion Monte Carlo
Source: J Chem Theory Comput. 2026 Jul 20;22(15):7693–702. doi: 10.1021/acs.jctc.6c00882 (PMC13472088; doi:10.1021/acs.jctc.6c00882)
Supplement: Supplementary file 1 [file ct6c00882_si_001.pdf]

Supplementary Information: Nodal error behind discrepancies between coupled cluster and diffusion Monte Carlo

S. Lambie[1], P. López Ríos[1], D. Kats[1], and A. Alavi[1,2,*]

[1] *Max Planck Institute for Solid State Research, Heisenbergstraße 1, 70569 Stuttgart, Germany*

[2] *Yusuf Hamied Department of Chemistry, University of Cambridge, Lensfield Road, Cambridge CB2 1EW, United Kingdom*

[*] Email: a.alavi@fkf.mpg.de

June 19, 2026

S1 Additional coupled cluster results

Table S1: Density-fitted (DF) and heavy-atom only augmented (HA) CCSD(T) interaction energies for the AcOH dimer. 0.5CP-corrected results are reported, with the error being one half of the total CP correction.

In text-referral	Treatment	E_{int} [kcal mol^{-1}]
DF-CCSD(T)	HF/DF-aV5Z + MP2/DF-aV{Q,5}Z + ΔCCSD(T)/aV{T,Q}Z	−19.41(5)
HA-CCSD(T)	HF/aVQZ+ CCSD(T)/haV{T,Q}Z	−19.42(2)

Heavy atom augmented basis sets/HA-CCSD(T)

A widely-employed basis set in the calculation of non-covalent interactions is the so-called 'heavy atom augmented basis set' whereby augmentation functions are removed from the H atom basis sets, with reportedly minimal effect on the interaction energies.[1] Here, we show results that support this for the AcOH dimer.

Density fitted basis sets/DF-CCSD(T)

Density fitting (DF) is often used to reduce the memory and increase the speed of quantum chemical calculations. For all density fitted calculations carried out in this work, the JKFIT[2] and MPFIT[3] basis sets are used are at same cardinal number as the atomic orbital basis set.

S2 VMC and DMC calculations

S2.1 Trial wave functions and optimization

Our basic trial wave function is of the Slater-Jastrow (SJ) form,

$$\Psi_{\rm SJ}(\mathbf{R}) = e^{J(\mathbf{R})}\Psi_{\rm S}(\mathbf{R}) \ , \tag{1}$$

with

$$\Psi_{\rm S}(\mathbf{R}) = \det[\phi_i(\mathbf{r}_j^{\uparrow})]\det[\phi_i(\mathbf{r}_j^{\downarrow})] \ , \tag{2}$$

where $\mathbf{R}$ denotes the set of up- and down-spin electron position vectors $\{\mathbf{r}_j^{\uparrow}\}$ and $\{\mathbf{r}_j^{\downarrow}\}$, $\{\phi_i\}$ are spin-independent single-particle molecular orbitals and e^J is a Jastrow correlation factor. In our all-electron (AE) calculations we modify the orbitals close to the nuclei in order to impose the electron-nucleus Kato cusp conditions.[4]

S2.1.1 Jastrow factor

We use Jastrow factors including isotropic electron-electron, electron-nucleus, and electron-electron-nucleus terms,[5]

$$J = \sum_{i<j}^{N_{\rm e}} u_{P_{ij}}(r_{ij}) + \sum_{i}^{N_{\rm e}}\sum_{I}^{N_{\rm n}} \chi_{S_{iI}}(r_{iI}) + \sum_{i<j}^{N_{\rm e}}\sum_{I}^{N_{\rm n}} f_{T_{ijI}}(r_{ij}, r_{iI}, r_{jI}) \ , \tag{3}$$

which are expressed as natural-power expansions in the relevant inter-particle distances,[6]

$$\begin{aligned} u_P(r_{ij}) &= t(r_{ij}, L_u)\sum_{k=0}^{n_u} a_k^{(P)} r_{ij}^k \ , \\ \chi_S(r_{iI}) &= t(r_{iI}, L_\chi)\sum_{k=0}^{n_\chi} b_k^{(S)} r_{iI}^k \ , \\ f_T(r_{ij}, r_{iI}, r_{jI}) &= t(r_{iI}, L_f)t(r_{jI}, L_f)\sum_{k,l,m=0}^{n_f} c_{klm}^{(T)} r_{ij}^k r_{iI}^l r_{jI}^m \ , \end{aligned} \tag{4}$$

where $n_u = 8$, $n_\chi = 8$, and $n_f = 3$ are expansion orders, $\{a\}$, $\{b\}$, and $\{c\}$ are optimizable parameters, $L_u = 4.5$, $L_\chi = 4$, and $L_f = 4$ are fixed cut-off lengths, $t(r, L) = (1-r/L)^3\Theta_{\rm H}(L-r)$ is a cut-off function, and $\Theta_{\rm H}(x)$ is the Heaviside step function. Indices P_{ij}, S_{iI}, and T_{ijI} allow the use of different parameter sets for same- and opposite-spin electron pairs and for different atomic species on distinct molecules. Our Jastrow factors for the acetic acid monomer and dimer contain 196 free parameters, while those for the water, peptide, and water-peptide systems contain 136, 256, and 376 parameters, respectively.

S2.1.2 Backflow transformation

The Slater-Jastrow-backflow (SJB) wave function is of the form

$$\Psi_{\rm SJB} = e^{J(\mathbf{R})}\Psi_{\rm S}[\mathbf{X}(\mathbf{R})] \ , \tag{5}$$

where the quasiparticle coordinates $\{\mathbf{x}_i(\mathbf{R})\}$ are of the inhomogeneous form proposed by López Ríos *et al.*[7] including electron-electron, electron-nucleus, and electron-electron-nucleus terms,

$$\mathbf{x}_i = \mathbf{r}_i + \sum_{j\neq i}^{N} \eta_{P_{ij}}(r_{ij})\mathbf{r}_{ij} + \sum_{I}^{N_{\rm n}} \mu_{S_{ij}}(r_{iI})\mathbf{r}_{iI} + \sum_{j\neq i}^{N}\sum_{I}^{N_{\rm n}} \left[\Phi_{T_{ijI}}(r_{ij}, r_{iI}, r_{jI})\mathbf{r}_{ij} + \Theta_{T_{ijI}}(r_{ij}, r_{iI}, r_{jI})\mathbf{r}_{iI}\right] \ , \tag{6}$$

which, like the functions in the Jastrow factor, are expressed as natural-power expansions in the relevant inter-particle distances,

$$
\begin{aligned}
\eta_P(r) &= t_P(r, L_\eta) \sum_{k=0}^{n_\eta} c_k^{(P)} r^k \ , \\
\mu_S(r) &= t_S(r, L_\mu) \sum_{k=0}^{n_\mu} d_k^{(S)} r^k \ , \\
\Phi_T(r_{ij}, r_{iI}, r_{iJ}) &= t_T(r_{iI}, L_\Phi) t_T(r_{jI}, L_\Phi) \sum_{k,l,m=0}^{n_\Phi} \phi_{klm}^{(T)} r_{ij}^k r_{iI}^l r_{jI}^m \ , \\
\Theta_T(r_{ij}, r_{iI}, r_{iJ}) &= t_T(r_{iI}, L_\Phi) t_T(r_{jI}, L_\Phi) \sum_{k,l,m=0}^{n_\Phi} \theta_{klm}^{(T)} r_{ij}^k r_{iI}^l r_{jI}^m \ ,
\end{aligned} \tag{7}
$$

where $n_\eta = 8$, $n_\mu = 8$, and $n_\Phi = 2$ are expansion orders, $\{c\}$, $\{d\}$, $\{\phi\}$, and $\{\theta\}$ are optimizable parameters, and $L_\eta = 4$, $L_\mu = 4.5$, and $L_\Phi = 4.5$ are fixed cut-off lengths. Our backflow functions the acetic acid monomer and dimer contain 230 free parameters, while those for the water, peptide, and water-peptide systems contain 295, 557, and 819 parameters, respectively.

S2.1.3 Wave function optimization

We have optimized all of our wave functions using linear least-squares energy minimization[8,9] using a correlated-sampling approach with n_{opt} VMC-generated real-space configurations. For the acetic acid monomer and dimer, and for the water, peptide, and water-peptide systems we have used $n_{\mathrm{opt}} = 10^6$ for Slater-Jastrow wave functions. As detailed in Section S2.3, our Slater-Jastrow-backflow wave functions have been optimized with a number of configurations chosen so as to keep the optimization uncertainty under a threshold; we have used $n_{\mathrm{opt}} = 10^7$ for the acetic acid monomer and dimer, and $n_{\mathrm{opt}} = 10^7$, 10^6, and 7.5×10^6 respectively for the water, peptide, and water-peptide systems.

S2.2 DMC calculation details

S2.2.1 DMC time step convergence of SJ-DMC results

Time-step bias is a smooth function of the DMC time step which is linear in τ as $\tau \to 0$, and it is often negligible in energy differences due to cancellation of errors. However care must be taken to verify the range of linearity and/or the degree of cancellation on a case-by-case basis, noting that the choice of localization scheme significantly affects time-step dependence.

We have chosen to use the T-move localization scheme in our calculations to avoid the beyond-first-order behavior seen for the determinant localization approximation (DLA) in the time-step bias plots of the supplementary information of Ref. 10. It should be noted that for all systems in the S66 set, the "typical" time-step range of $\tau = 0.01$–0.04 a.u. suggested for ECP systems in Ref. 11 appears falls outside the regime where total energies can be regarded to be linear functions of τ, and therefore this range suggestion should not be followed blindly when the DLA scheme is used.

In Fig. S1 we plot the time-step dependence of the total DMC energies obtained for the AcOH monomer and dimer, and Fig. S2 shows the time-step dependence of the total DMC energies obtained for the water, peptide, and water-peptide systems. Our ECP SJ-DMC and ECP SJB-DMC results have been obtained by linear extrapolation to zero time step of the energies obtained at $\tau = 0.004$ and 0.016 a.u., for efficiency,[12,13] and these energies and fits are shown in Figs. S1 and S2, along with additional DMC energies at other time steps to provide visual confirmation that the ECP DMC energy is linear in the time step for the ECP systems.

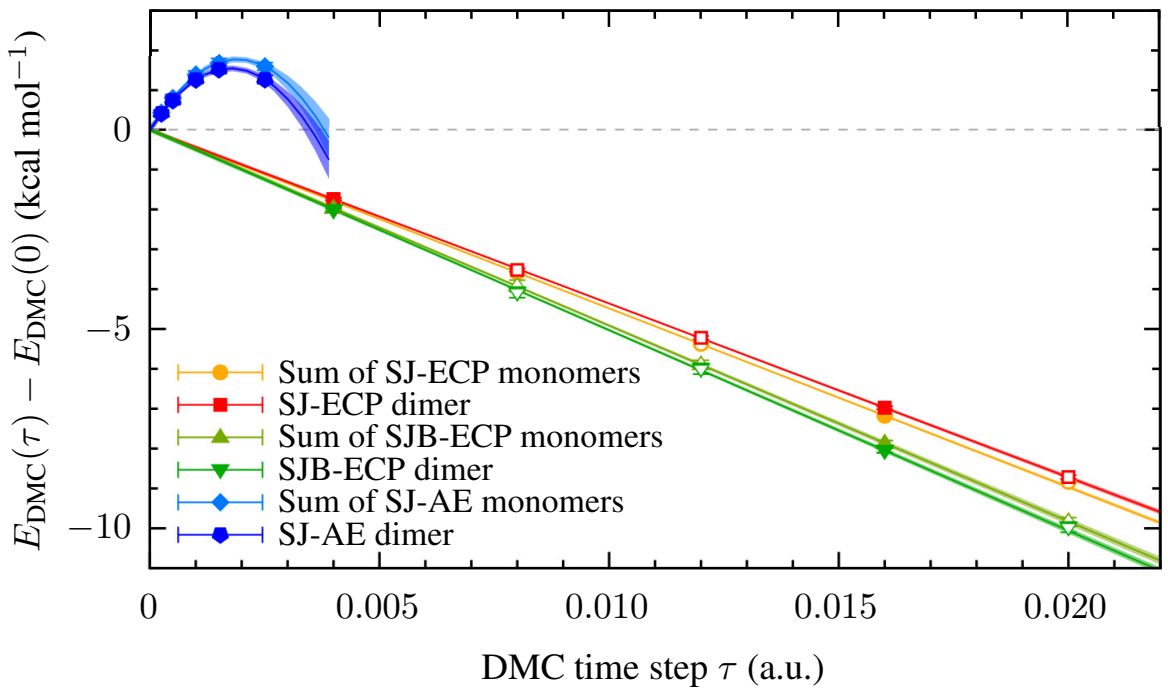

Figure S1: Time-step bias on the ECP SJ-DMC and ECP SJB-DMC total energy of the AcOH dimer and on twice the total energy of the AcOH monomer, along with AE SJ-DMC results for comparison. Solid symbols represent data points used in the corresponding fits, while open symbols are ignored in fits and shown to verify linear trends only. Lines represent fits to the data, and shaded areas around them represent 95% confidence intervals on the fit values, while errorbars represent 95% confidence intervals around data points.

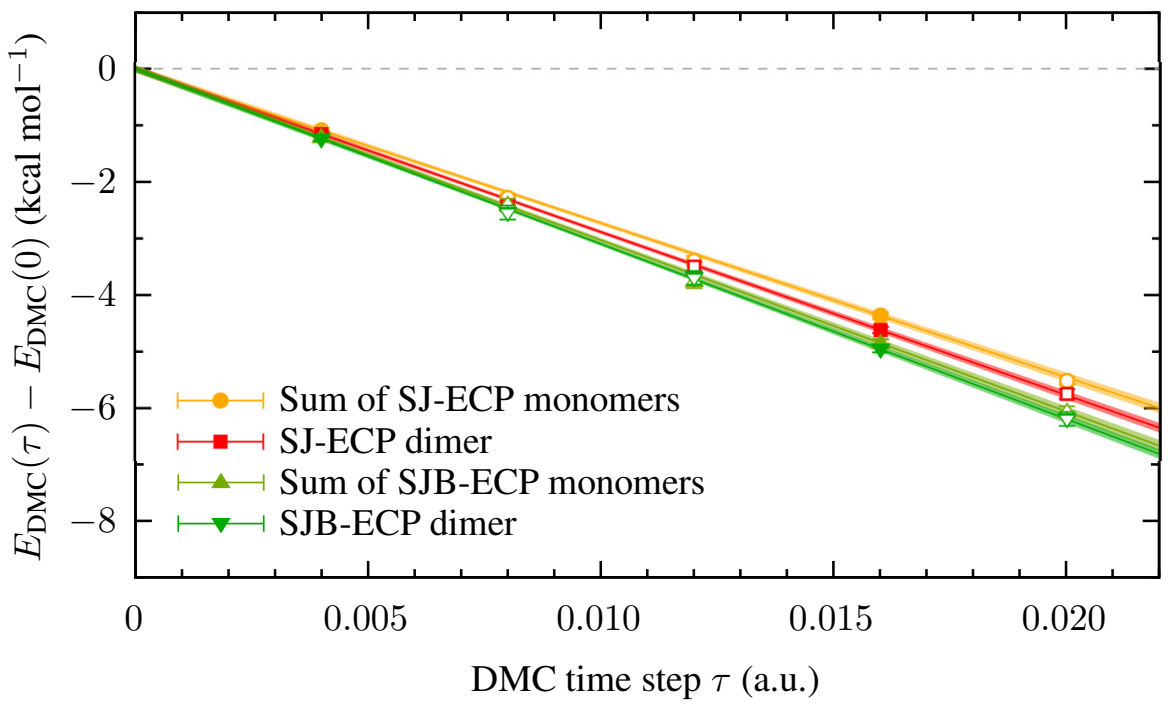

Figure S2: Time-step bias on the ECP SJ-DMC and ECP SJB-DMC total energy of the water-peptide system and on the sum of the total energies of the water and peptide monomers. Solid symbols represent data points used in the corresponding fits, while open symbols are ignored in fits and shown to verify linear trends only. Lines represent fits to the data, and shaded areas around them represent 95% confidence intervals on the fit values, while errorbars represent 95% confidence intervals around data points.

Note that the time steps for our ECP calculations were chosen so that DMC energies using the DLA scheme, should we have chosen to run them, would have been linearly extrapolatable to zero time step; if we had targeted the T-move scheme alone from the outset we should have been able to use significantly larger time steps.

For the AcOH monomer and dimer, Fig. S1 also shows AE SJ-DMC energies, which we extrapolate to zero time step by fitting all five data points to a second-order polynomial, and we use this result to test the accuracy of the ECP approximation. The two-point linear extrapolation of the ECP SJ-DMC results gives an interaction energy of $-20.13(6)$ kcal mol^{-1}, within uncertainty of the quadratic extrapolation of the AE SJ-DMC energies of $-20.29(16)$ kcal mol^{-1}, and we conclude that the ECP approximation does not introduce a significant error.

S2.2.2 DMC population control bias

We have used a target DMC population of 65536 walkers at the smallest time step for each system, and have keep the population proportional to $1/\tau$ at other time steps in order to remove the (likely negligible) population control bias from our results.[11]

S2.3 SJB-DMC optimization uncertainty

The uncertainty introduced by the stochastic optimization of wave function parameters is typically ignored in VMC and DMC calculations. This is partly justified in some cases: the error in the VMC energy is quadratic in the error in the optimized parameter set when energy minimization is used, while the SJ-DMC energy of all-electron systems at zero time step is independent of the parameter values. However VMC energies of variance-minimized wave functions,[14,15] VMC expectation values of operators other than the Hamiltonian,[16] and beyond-SJ DMC energies are affected by errors linear in the error in the optimized parameter set; obtaining accurate values for these requires converging the results with respect to sample size.[14,15]

S2.3.1 Dependence of the optimization uncertainty on the VMC-based optimization sample size

In correlated-sampling optimization, one generates $n_{\rm opt}$ random real-space electronic configurations $\{\mathbf{R}_i\}_{i=1}^{n_{\rm opt}}$ distributed according to an initial wave function $\Psi(\mathrm{p};\mathbf{R})$, where $\mathbf{p}$ are the wave function parameters. The local energy is evaluated at these configurations, $E_{\rm L}(\mathbf{p},\mathbf{R}_i) = \Psi^{-1}(\mathbf{p},\mathbf{R}_i)\hat{H}(\mathbf{R}_i)\Psi(\mathbf{p},\mathbf{R}_i)$, and the resulting VMC energy $E_{\rm VMC}(\mathbf{p})$ is computed as the average of the local energies, which has a standard error proportional to $n_{\rm opt}^{-1/2}$. The variational energy is then minimized by varying $\mathbf{p}$ while keeping the configurations $\{\mathbf{R}_i\}_{i=1}^{n_{\rm opt}}$ fixed. The optimizer will clearly only be able to locate the parameters that minimize the variational energy to the degree that the statistical uncertainty on the variational energy allows, and thus the the parameter set $\mathbf{p}'$ resulting from the optimization procedure is a random vector which follows a probability distribution of spread determined by that of the variational energy. At sufficiently large sample sizes, the central limit theorem applies to both $E_{\rm VMC}(\mathbf{p})$ and $\mathbf{p}'$, and by error propagation to leading order, the uncertainty on $\mathbf{p}'$ is proportional to that in $E_{\rm VMC}(\mathbf{p})$, which in turn makes it proportional to $n_{\rm opt}^{-1/2}$.

The DMC energy is a function of the optimized wave function parameters, $E_{\rm DMC}(\mathbf{p}')$, and again by error propagation to leading order, the contribution from the uncertainty on $\mathbf{p}'$ to the uncertainty on $E_{\rm DMC}(\mathbf{p}')$ is proportional to $n_{\rm opt}^{-1/2}$. Thus, the optimization uncertainty on the SJB-DMC energy is indirectly dependent on the number of real-space configurations $n_{\rm opt}$ used in the VMC-based correlated-sampling optimization process.

S2.3.2 Evaluating the optimization uncertainty

The most direct, brute-force way of obtaining the optimization uncertainty, $\sigma_{\rm opt}$, for any given system is to perform multiple independent random instances of wave function optimization each followed by a DMC run, and evaluating $\sigma_{\rm opt}$ as the standard deviation of the resulting DMC energies. For the systems in this work we perform 20 such independent random instances, and we perform a single DMC run at $\tau = 0.016$ a.u. under the assumption that $\sigma_{\rm opt}$ is independent of time step, which we verify below.

In order to ascertain that $\sigma_{\rm opt}$ is a well-behaved function of $n_{\rm opt}$, we have evaluated the optimization uncertainty of the AcOH monomer, $\sigma_{\rm opt}^{\rm AcOH}$, at each of four sample sizes $n_{\rm opt}$ ranging between 10^4 and 10^6. Given that the interaction between the AcOH monomers in the dimer is weak, we take the optimization uncertainty for the AcOH dimer, $\sigma_{\rm opt}^{\rm (AcOH)_2}$, to be very close to $\sqrt{2}$ times that of the monomer at the same $n_{\rm opt}$. We have verified this at $n_{\rm opt} = 10^5$, where we obtain an uncertainty ratio of $\sigma_{\rm opt}^{\rm (AcOH)_2}/\sigma_{\rm opt}^{\rm AcOH} = 1.72 \pm 0.54$, which well within one standard deviation from $\sqrt{2}$. We take the total optimization uncertainty on the interaction energy of the AcOH dimer to be $\sigma_{\rm opt} = \sqrt{(2\sigma_{\rm opt}^{\rm AcOH})^2 + {\sigma_{\rm opt}^{\rm (AcOH)_2}}^2} = \sqrt{6}\sigma_{\rm opt}^{\rm AcOH}$, so our target optimization uncertainty for the interaction energy of $\sigma_{\rm opt} = 0.071$ kcal mol^{-1} translates into a target $\sigma_{\rm opt}^{\rm AcOH} \approx 0.029$ kcal mol^{-1}.

We have also tested the time-step dependence of the optimization uncertainty by runnning SJB-DMC calculations at $\tau = 0.004$ a.u. for each of the 20 SJB wave functions optimized at $n_{\rm opt} = 10^5$, and we plot the corresponding linear extrapolations in Fig. S3. The standard deviation of the SJB-DMC energies extrapolated to $\tau = 0$ is 0.14 kcal mol^{-1}, which is about half that at $\tau = 0.016$ a.u. of 0.26 kcal mol^{-1}. We conclude that approximating the optimization uncertainty at $\tau = 0$ by the value obtained at $\tau = 0.016$ a.u. provides a safe overestimation of its true value, and we apply this approximation throughout.

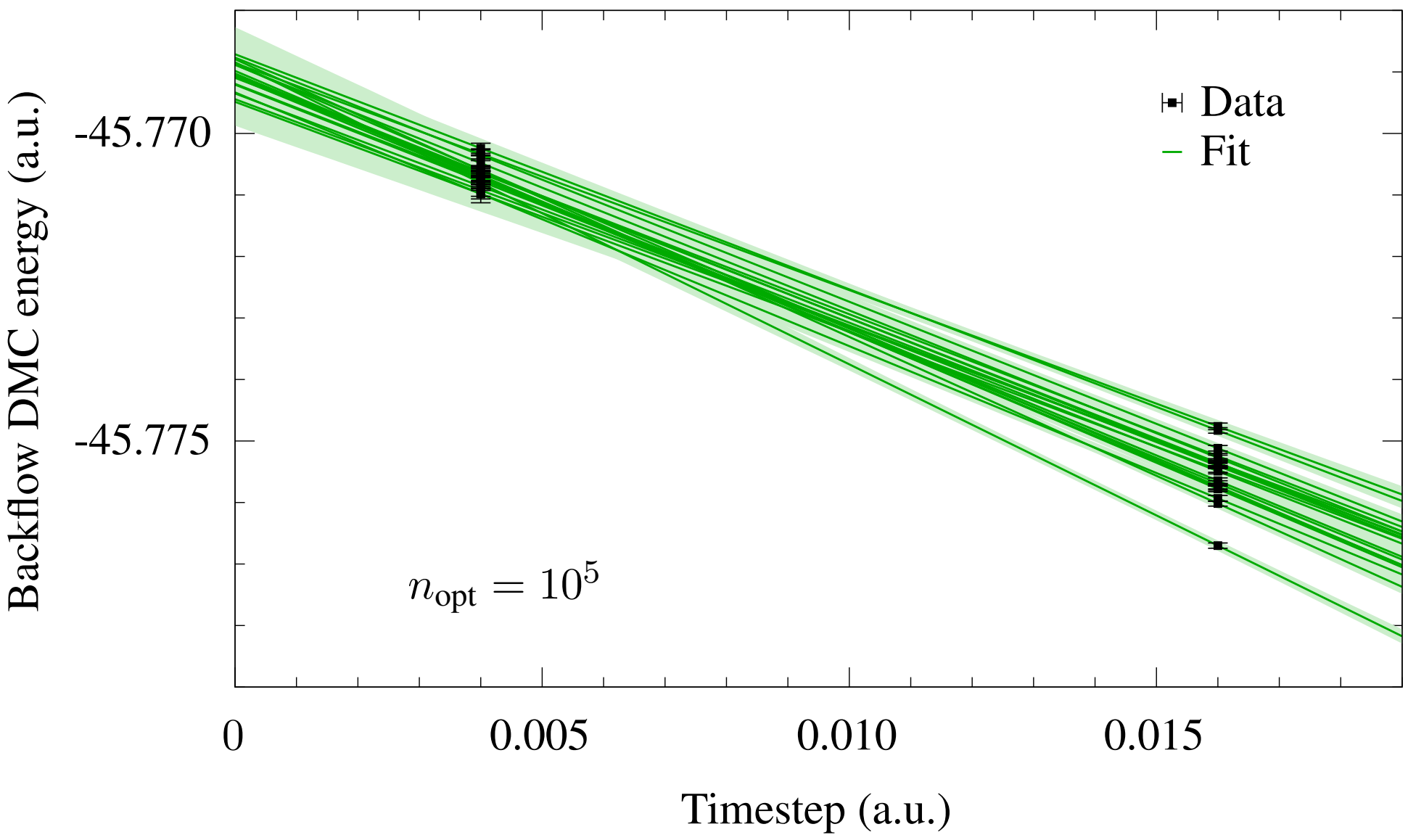

Figure S3: ECP SJB-DMC energy as a function of time step for 20 independent random instances of wave function optimization at sample size $n_{\rm opt} = 10^5$. Lines are linear fits to the data, and shaded areas around them represent 95% confidence intervals on fit values, while errorbars represent 95% confidence intervals around data points.

We compute the optimization sample size by linear extrapolation in log-log scale using the formula $\log \sigma_{\rm opt} = \alpha - 1/2 \log n_{\rm opt}$. For the AcOH molecule we perform a least-squares fit to this formula using the result from all four sample sizes, and for other systems where we only perform tests at $n_{\rm opt} = 30\,000$ we simply substitute the values of $\sigma_{\rm opt}$ and $n_{\rm opt}$ into the formula and solve for α.

For the AcOH monomer and dimer we use $n_{\rm opt} = 10^7$, which yields an optimization uncertainty on the interaction energy at $n_{\rm opt} = 10^7$ of $\sigma_{\rm opt} = 0.060$ kcal mol^{-1}, below our target of 0.071 kcal mol^{-1}. For the water, peptide, and water-peptide systems we use $n_{\rm opt} = 10^7$, 10^6, and 7.5×10^6, which yield optimization uncertainties on the total energies of 0.023, 0.042, and 0.049 kcal mol^{-1}, respectively, and an optimization uncertainty on the interaction energy of $\sigma_{\rm opt} = 0.069$ kcal mol^{-1}, again below our target of 0.071 kcal mol^{-1}.

S2.3.3 DMC results

The DMC energies from which the final results have been obtained can be found in Table S2. Also shown is the energy correction attributable to the use of backflow, $\Delta_{\rm BF} = E^{\rm (DMC)}_{\rm SJB} - E^{\rm (DMC)}_{\rm SJ}$, which compares very favourably with the corrections obtained using other nodal optimization techniques; for example note that the antisymmetric geminal power (AGP) ansatz used in Ref. 17 yield corrections $\Delta_{\rm AGP} = -8.0(3)$ and $-5.2(2)$ mHa for the $\rm (AcOH)_2$ and water-peptide dimers, respectively, a factor of 4–5 smaller than the values of $\Delta_{\rm BF}$ shown in Table S2.

In the spirit of variance-matching methods,[18–21] it is relevant to assess how well-balanced our monomer and dimer SJB wave functions are by looking at their VMC variances. In Table S2

we show the VMC variance of the local energy, $\sigma^{2\,(\mathrm{VMC})}_{\mathrm{SJB}}$, for each monomer, sum of monomers, and dimer, evaluated with the tail-regression estimator[22] using the TREAT utility[23] to ensure that the resulting confidence intervals are well-defined. We find that the variance of $(\mathrm{AcOH})_2$ is slightly lower than twice the variance of AcOH, while the variance of the water-peptide system is marginally higher than the sum of the variances of the water and peptide monomers. In both cases, if wave function balance were to be improved, be it by improving the wave functions of the higher-variance systems or by deliberately worsening those of the lower-variance systems in a controlled manner, the SJB-DMC interaction energies would shift towards improved agreement with CC, leaving the conclusions of our present study unchanged.

System	$E_{SJ}^{(DMC)}$ (Ha)	$E_{SJB}^{(DMC)}$ (Ha)	Δ_{BF} (mHa)	n_{opt}	σ_{opt} (mHa)	$\sigma_{SJB}^{2\ (VMC)}$ (Ha^2)
$(AcOH)_2$						
- Monomer	−45.752954(15)	−45.769353(23)	−16.40(3)	10^7	0.039	0.707(1)
- 2 × monomer	−91.505908(30)	−91.538706(46)	−32.80(6)	–	0.078	1.414(2)
- Dimer	−91.537980(37)	−91.570021(47)	−32.04(6)	10^7	0.055	1.398(3)
Water-peptide						
- Water	−17.201852(25)	−17.208468(24)	−6.62(4)	10^7	0.037	0.2091(7)
- Peptide	−47.151055(43)	−47.169548(45)	−18.49(6)	10^6	0.067	0.742(2)
- Sum monomers	−64.352907(50)	−64.378016(51)	−25.11(7)	–	0.077	0.951(2)
- Dimer	−64.366296(45)	−64.390954(44)	−24.66(6)	7.5×10^7	0.078	0.961(2)

Table S2: Zero time step DMC energies $E_{SJ}^{(DMC)}$ and $E_{SJB}^{(DMC)}$, backflow corrections to the DMC energy Δ_{BF}, optimization sample sizes n_{opt}, optimization uncertainties σ_{opt}, and VMC variances $\sigma_{SJB}^{2\ (VMC)}$ for the ECP molecules and dimers considered in this study. The $E_{SJB}^{(DMC)}$ figures shown do not include the contribution from σ_{opt} in the uncertainties. Note that, by contrast with results presented elsewhere in this work, these results are in atomic units and the uncertainties shown are 68% (1-sigma) confidence intervals, as typically used for raw data in quantum Monte Carlo contexts.

S3 Geometries

AcOH dimer

```
16

C   -1.061709204   1.297140572   0.292060003
O   -0.358161116   2.270458613   0.531812668
O   -0.589303516   0.094917758   0.003788813
H    0.404435659   0.127722621   0.018411838
C   -2.558427798   1.342549823   0.296257320
H   -2.895997978   2.347464002   0.518316340
H   -2.932889278   1.022390451  -0.672995551
H   -2.937211960   0.644910433   1.039557084
C    2.789348447   1.108419242   0.271183758
O    2.085730082   0.135104754   0.031396156
O    2.316922113   2.310854630   0.558962229
H    1.323133573   2.277956396   0.544561725
C    4.286060895   1.062516496   0.269219363
H    4.623640459   0.061197303   0.031693868
H    4.667559440   1.772869435  -0.460249535
H    4.657577206   1.365211013   1.245274724
```

Water-peptide

```
 15

 O   -0.392018453  -0.384718737   0.076071325
 H   -0.911460851   0.413812040   0.177648774
 H    0.524903820  -0.068484694   0.090511364
 C    2.197705212  -2.245403490  -0.230313251
 H    2.847668055  -3.106515367  -0.363228638
 H    1.516729242  -2.167931431  -1.074178526
 H    1.584688314  -2.384199477   0.656695112
 C    2.952437290  -0.947390613  -0.097719744
 O    2.375721843   0.127904243   0.058868998
 N    4.303070413  -1.044893297  -0.162337713
 H    4.704022041  -1.955427276  -0.291852811
 C    5.171312525   0.107077163  -0.052894631
 H    4.534818395   0.975377606   0.081889976
 H    5.836902032   0.015621965   0.803198250
 H    5.765778248   0.236497652  -0.955153818
```

References

[1] Marshall, M. S.; Sears, J. S.; Burns, L. A.; Breédas, J.-L.; Sherrill, C. D. An error and efficiency analysis of approximations to Møller-Plesset perturbation theory. J. Chem. Theory Comput. **2010**, 6, 3681–3687.

[2] Weigend, F. A fully direct RI-HF algorithm: Implementation, optimised auxiliary basis sets, demonstration of accuracy and efficiency. Phys. Chem. Chem. Phys. **2002**, 4, 4285–4291.

[3] Weigend, F.; Köhn, A.; Hättig, C. Efficient use of the correlation consistent basis sets in resolution of the identity MP2 calculations. J. Chem. Phys. **2002**, 116, 3175–3183.

[4] Ma, A.; Towler, M. D.; Drummond, N. D.; Needs, R. J. Scheme for adding electron–nucleus cusps to Gaussian orbitals. J. Chem. Phys. **2005**, 122, 224322.

[5] López Ríos, P.; Seth, P.; Drummond, N. D.; Needs, R. J. Framework for constructing generic Jastrow correlation factors. Phys. Rev. E **2012**, 86, 036703.

[6] Drummond, N. D.; Towler, M. D.; Needs, R. J. Jastrow correlation factor for atoms, molecules, and solids. Phys. Rev. B **2004**, 70, 235119.

[7] López Ríos, P.; Ma, A.; Drummond, N. D.; Towler, M. D.; Needs, R. J. Inhomogeneous backflow transformations in quantum Monte Carlo calculations. Phys. Rev. E **2006**, 74, 066701.

[8] Toulouse, J.; Umrigar, C. J. Optimization of quantum Monte Carlo wave functions by energy minimization. J. Chem. Phys. **2007**, 126, 084102.

[9] Umrigar, C. J.; Toulouse, J.; Filippi, C.; Sorella, S.; Hennig, R. G. Alleviation of the Fermion-Sign Problem by Optimization of Many-Body Wave Functions. Phys. Rev. Lett. **2007**, 98, 110201.

[10] Shi, B. X.; Pia, F. D.; Al-Hamdani, Y. S.; Michaelides, A.; Alfé, D.; Zen, A. Systematic discrepancies between reference methods for non-covalent interactions within the S66 dataset. J. Chem. Phys. **2025**, 162, 144107.

[11] Needs, R. J.; Towler, M. D.; Drummond, N. D.; López Ríos, P.; Trail, J. R. Variational and diffusion quantum Monte Carlo calculations with the CASINO code. J. Chem. Phys. **2020**, 152, 154106.

[12] Lee, R. M.; Conduit, G. J.; Nemec, N.; López Ríos, P.; Drummond, N. D. Strategies for improving the efficiency of quantum Monte Carlo calculations. Phys. Rev. E **2011**, 83, 066706.

[13] Vrbik, J.; Rothstein, S. M. Optimal spacing and weights in diffusion Monte Carlo. International Journal of Quantum Chemistry **1986**, 29, 461–468.

[14] Haupt, J. P.; Hosseini, S. M.; López Ríos, P.; Dobrautz, W.; Cohen, A.; Alavi, A. Optimizing Jastrow factors for the transcorrelated method. J. Chem. Phys. **2023**, 158, 224105.

[15] Filip, M.-A.; Christlmaier, E. M. C.; Haupt, J. P.; Kats, D.; López Ríos, P.; Alavi, A. Deterministic optimization of Jastrow factors. J. Chem. Phys. **2025**, 163, 084107.

[16] Spink, G. G.; López Ríos, P.; Drummond, N. D.; Needs, R. J. Trion formation in a two-dimensional hole-doped electron gas. Phys. Rev. B **2016**, 94, 041410.

[17] Nakano, K.; Shi, B. X.; Alfè, D.; Zen, A. Assessing the impact of nodal surface optimization in fixed-node diffusion Monte Carlo on non-covalent interactions. arXiv **2026**, 2604.04329.

[18] Robinson, P. J.; Pineda Flores, S. D.; Neuscamman, E. Excitation variance matching with limited configuration interaction expansions in variational Monte Carlo. J. Chem. Phys. **2017**, 147, 164114.

[19] Dash, M.; Feldt, J.; Moroni, S.; Scemama, A.; Filippi, C. Excited States with Selected Configuration Interaction-Quantum Monte Carlo: Chemically Accurate Excitation Energies and Geometries. J. Chem. Theory Comput. **2019**, 15, 4896–4906.

[20] Dash, M.; Moroni, S.; Filippi, C.; Scemama, A. Tailoring CIPSI Expansions for QMC Calculations of Electronic Excitations: The Case Study of Thiophene. J. Chem. Theory Comput. **2021**, 17, 3426–3434.

[21] Fu, W.; Ren, W.; Chen, J. Variance extrapolation method for neural-network variational Monte Carlo. Mach. Learn.: Sci. Technol. **2024**, 5, 015016.

[22] López Ríos, P.; Conduit, G. J. Tail-regression estimator for heavy-tailed distributions of known tail indices and its application to continuum quantum Monte Carlo data. Phys. Rev. E **2019**, 99, 063312.

[23] López Ríos, P. TREAT: tail-regression estimator analysis toolbox. 2019; `https://github.com/plopezrios/treat`.
